# Supplementary material for: AI-assisted radiologists vs. standard double reading for rib fracture detection on CT images: A real-world clinical study
Source: PLoS One. 2025 Jan 24;20(1):e0316732. doi: 10.1371/journal.pone.0316732 (PMC11760585; doi:10.1371/journal.pone.0316732)
Supplement: S1 Table — (DOCX) [file pone.0316732.s001.docx]

**S1 Table. Information of radiologists.**

| **Radiologists number** | **Gender** | **Years of practice** |
| --- | --- | --- |
| Residents |  |  |
| R1 | Male | 3 |
| R2 | Female | 2 |
| Attending radiologists |  |  |
| R3 | Female | 8 |
| R4 | Female | 7 |
| R5 | Female | 8 |
| R6 | Female | 6 |
| R7 | Female | 4 |
| Senior radiologists |  |  |
| R8 | Male | 14 |
| R9 | Female | 16 |
| R10 | Female | 11 |
| R11 | Male | 20 |
| R12 | Male | 23 |
| R13 | Male | 19 |
| R14 | Female | 21 |
| R15 | Female | 18 |

Note—R1-R15 represents Radiologists 1-15.
