# Supplementary figures and images for: AI-assisted radiologists vs. standard double reading for rib fracture detection on CT images: A real-world clinical study
Source: PLoS One. 2025 Jan 24;20(1):e0316732. doi: 10.1371/journal.pone.0316732 (PMC11760585; doi:10.1371/journal.pone.0316732)

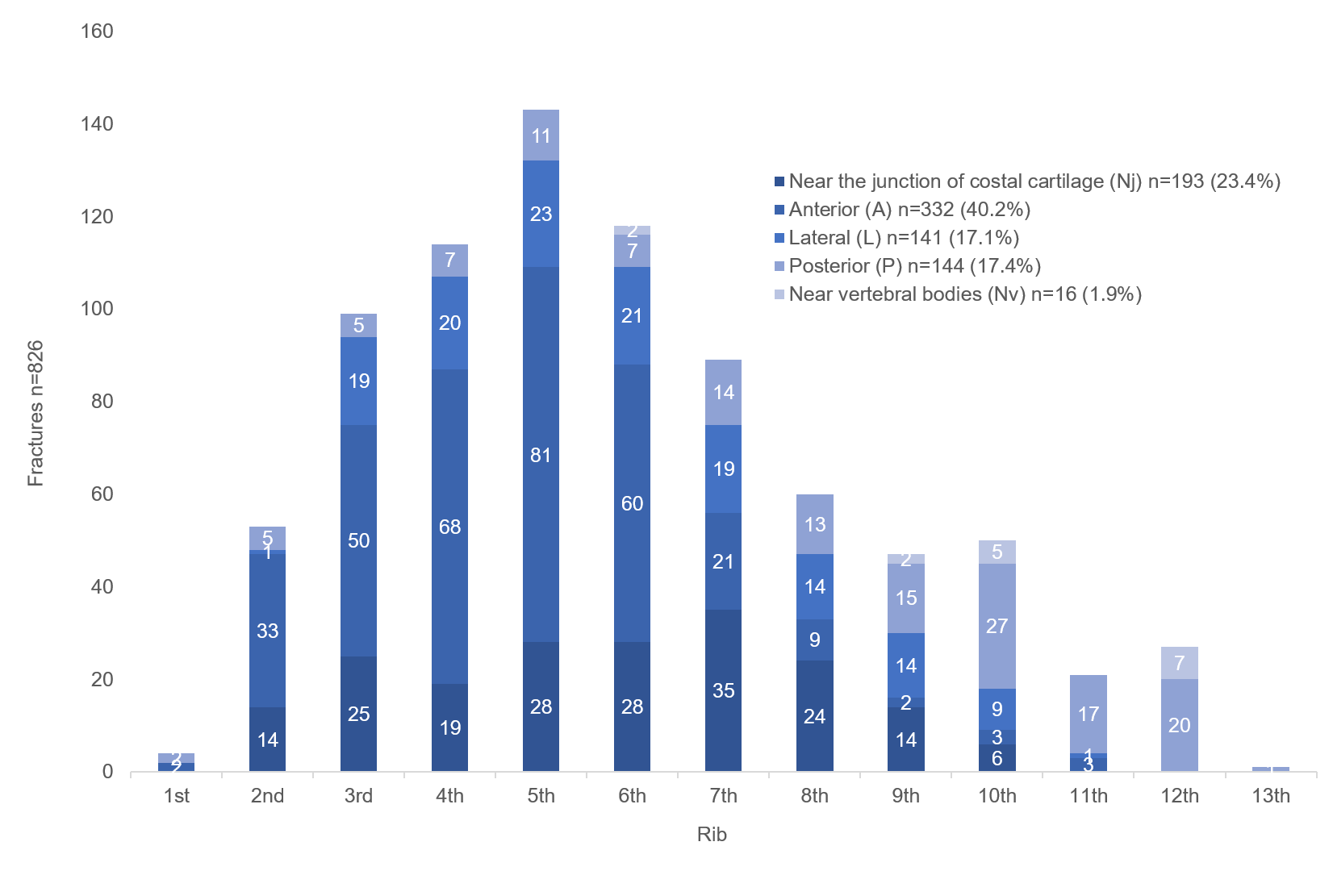

Supplement: S1 Fig — The most common fracture location was the region A (40.2%) followed by Nj (23.4%). The prevalence of RFs approximated a Gaussian distribution, with the apex appearing in the fifth rib. More than 56% of all RFs were between the 4th and 7th ribs. (Nj = near the junction of costal cartilage, A = anterior, L = lateral, P = posterior, Nv = near vertebral bodies). (TIF) [file pone.0316732.s002.tif]
