# Supplementary material for: AI-assisted radiologists vs. standard double reading for rib fracture detection on CT images: A real-world clinical study
Source: PLoS One. 2025 Jan 24;20(1):e0316732. doi: 10.1371/journal.pone.0316732 (PMC11760585; doi:10.1371/journal.pone.0316732)
Supplement: Attachment — Submitted filename: Response to Reviewers.docx [file pone.0316732.s003.docx]

Dear Editors and Reviewers,

Thank you for your thorough review and insightful comments on our manuscript. We have carefully considered each point and have provided our responses below:

**Response to Reviewer #1:**

Thank you for your positive comments on our manuscript. We are gratified to hear that you found the study interesting and well-executed. Your comments encourage us and affirm the value of our research.

**Response to Reviewer #2:**

Thank you for your insightful comments. We have addressed each of your points as follows:

- **Background Information on Radiologists:**

We appreciate your suggestion to include more background information on the radiologists involved in the study. We have expanded the relevant section in the main manuscript (Materials and Methods; 4. Image interpretation, lines 5-9) to include their years of practice, which we believe better demonstrates the utility of AI. The updated sentence now reads: " AII primary reports were conducted by 2 residents (2nd and 3rd year of residency; female, 1) and 5 attending radiologists (4 to 8 years of experience; mean, 6.6±1.7 years of experience; female, 5), with secondary readings performed by senior radiologists with over 10 years of experience (See S1 Table for details)." Additionally, we have included more comprehensive background details in the supplementary material (please see S1 Table). Additional details are provided in the supplementary material (S1 Table). While we agree that stratification by years of practice is valuable, the size constraints of our study currently limit the feasibility of conducting such an analysis. We hope to explore this issue with greater rigor in future endeavors, specifically through larger-scale, multicenter studies.

- **Pathologies Associated with Rib Fractures:**

We have heeded your advice and expanded our analysis to include associated pathologies. Data on the incidence of pneumothorax, pulmonary contusions, and hemothorax have been added to the "Materials and Methods 1. Dataset collection and access" section. The updated sentence reads: "Intrathoracic injuries associated with RFs included pneumothorax (5.3%, 10 of 188), pulmonary contusions (2.1%, 4 of 188), and hemothorax (1.1%, 2 of 188)." This addition is intended to provide a more comprehensive clinical context, aligning with the importance you have highlighted. We would also like to express our gratitude for your insightful proposal. The inclusion of other pathologic changes associated with rib fractures (RFs) more fully reflects the clinical conditions of the patients in our study. As the data indicate, the incidence of pathologies associated with RFs in this study is roughly the same as the results of some of the previous studies [1], but slightly lower than others [2]. While, the distribution of fracture characteristics, as shown in S2 Fig, is generally consistent with previous researches [3,4].

- **CT Scanner and Image Viewing Software and Monitor Specifications:**

We have included the specifications of the image viewing software and computer system used by the radiologists in the "Materials and Methods 1. Dataset collection and access" section. The updated sentence reads: “All images were reviewed through the picture archiving and communication system (PACS) (3.0.30500.213; Hinacom Software and Technology, Beijing, China) on a Windows 7 system (Microsoft Corporation, Redmond, Washington, United States) using professional radiographic monitors (JUSHA-M52C, JUSHA-M53AA, JUSHA-M33B and JUSHA-M33D; Nanjing Jusha Display Technology, Nanjing, China).”

Additionally, the specifications of the CT scanners are detailed: “Scans were performed on two Siemens CT scanners (Emotion 16 and SOMATOM Definition Flash) with a 120 kV tube voltage and automated dose modulation….”

- **Data from Single Read CT Scans:**

Regrettably, the collection of single read data was not feasible within the scope of our current study design. Our aim was to assess the performance of radiologists using AI assistance in a real-world clinical environment, which led us to integrate target patient images into the daily workflow through the PACS system. Given the full integration of AI-assisted diagnostic technology into our department's rib fracture diagnosis protocol, conducting single radiologist reads without AI assistance would not be practical without the radiologists' awareness, as it would deviate from our standard clinical practice. We acknowledge the importance of comparing single radiologist diagnosis with AI-assisted diagnosis, and we are aware of existing literature on this topic. Studies have consistently demonstrated that the incorporation of AI assistance in radiological diagnosis leads to a substantial enhancement in the sensitivity of single radiologists for rib fractures, concurrently preserving diagnostic specificity. The reported increase in sensitivity varies depending on the study design, ranging from 9.5% to 23.9% [5-7]. This underscores the potential clinical value of AI in enhancing diagnostic accuracy, especially in settings where radiologist resources may be limited, such as rural areas. We appreciate your guidance and will consider incorporating single read data in future prospective studies to further explore the impact of AI in various clinical scenarios, including those you've highlighted.

- **Time Required for Double Read vs. AI-Assisted Reads:**

We agree that evaluating diagnostic time is a valuable research direction. Prior studies have shown that AI assistance significantly reduces diagnostic time for radiologists, with savings ranging from 73.9 seconds to 771 seconds depending on study conditions [5,7]. As a retrospective study, we were unable to directly measure diagnostic time. This limitation is acknowledged in the "Discussion" section, specifically in the penultimate paragraph where we discuss the limitations of our study. The updated content is as follows: " Third, since this is a retrospective study, the time spent on diagnosis cannot be calculated. Previous studies have consistently demonstrated the ability of AI to shorten the reading time of radiologist in diagnosing RFs in chest CT. With the aid of AI, report time savings ranged from approximately 73.9 seconds to 771 seconds, depending on the study conditions..."

**Reference:**

[1] Tignanelli CJ, Rix A, Napolitano LM, Hemmila MR, Ma S, Kummerfeld E. Association Between Adherence to Evidence-Based Practices for Treatment of Patients With Traumatic Rib Fractures and Mortality Rates Among US Trauma Centers. JAMA Netw Open. 2020 Mar 2;3(3):e201316.

[2] Nummela MT, Bensch FV, Pyhältö TT, Koskinen SK. Incidence and Imaging Findings of Costal Cartilage Fractures in Patients with Blunt Chest Trauma: A Retrospective Review of 1461 Consecutive Whole-Body CT Examinations for Trauma. Radiology. 2018 Feb;286(2):696-704. doi: 10.1148/radiol.2017162429.

[3] Fukushima K, Kambe M, Aramaki Y, Ichikawa Y, Isshiki Y, Nakajima J, Sawada Y, Oshima K. Evaluation of injury threshold from the number of rib fracture for predicting pulmonary injuries in blunt chest trauma. Heliyon. 2023 Apr 6;9(4):e15278.

[4] Barber I, Perez-Rossello JM, Wilson CR, Kleinman PK. The yield of high-detail radiographic skeletal surveys in suspected infant abuse. Pediatr Radiol 2015;45(1):69-80.

[5] Zhou QQ, Wang J, Tang W, Hu ZC, Xia ZY, Li XS, et al. Automatic Detection and Classification of Rib Fractures on Thoracic CT Using Convolutional Neural Network: Accuracy and Feasibility. Korean J Radiol. 2020;21(7):869-879.

[6] Wu M, Chai Z, Qian G, Lin H, Wang Q, Wang L, et al. Development and Evaluation of a Deep Learning Algorithm for Rib Segmentation and Fracture Detection from Multicenter Chest CT Images. Radiol Artif Intell. 2021;3(5):e200248.

[7] Jin L, Yang J, Kuang K, Ni B, Gao Y, Sun Y, et al. Deep-learning-assisted detection and segmentation of rib fractures from CT scans: Development and validation of FracNet. EBioMedicine. 2020;62:103106.

We hope that these revisions adequately address your concern and strengthens the relevance of our study to clinical practice. We are committed to ensuring that our work is thorough and clinically relevant.

Thank you again for your constructive feedback.
